# The value of standards for health datasets in artificial intelligence-based applications

**DOI:** 10.1038/s41591-023-02608-w

**Published:** 2023-10-26

**Authors:** Anmol Arora, Joseph E. Alderman, Joanne Palmer, Shaswath Ganapathi, Elinor Laws, Melissa D. McCradden, Lauren Oakden-Rayner, Stephen R. Pfohl, Marzyeh Ghassemi, Francis McKay, Darren Treanor, Negar Rostamzadeh, Bilal Mateen, Jacqui Gath, Adewole O. Adebajo, Stephanie Kuku, Rubeta Matin, Katherine Heller, Elizabeth Sapey, Neil J. Sebire, Heather Cole-Lewis, Melanie Calvert, Alastair Denniston, Xiaoxuan Liu

**Affiliations:** 1 School of Clinical Medicine, University of Cambridge, Cambridge, UK; 2 Institute of Inflammation and Ageing, College of Medical and Dental Sciences, University of Birmingham, Birmingham, UK; 3 University Hospitals Birmingham NHS Foundation Trust, Birmingham, UK; 4 National Institute for Health and Care Research Birmingham Biomedical Research Centre, University of Birmingham, Birmingham, UK; 5 Sandwell and West Birmingham Hospitals NHS Trust, Birmingham, UK; 6 Department of Bioethics, The Hospital for Sick Children, Toronto, Ontario Canada; 7 Genetics and Genome Biology, Peter Gilgan Centre for Research and Learning, Toronto, Ontario Canada; 8 Dalla Lana School of Public Health, Toronto, Ontario Canada; 9 The Australian Institute for Machine Learning, University of Adelaide, Adelaide, South Australia Australia; 10 Google Research, Mountain View, CA USA; 11 Department of Electrical Engineering and Computer Science, Massachusetts Institute of Technology, Cambridge, MA USA; 12 Institute for Medical Engineering & Science, Massachusetts Institute of Technology, Cambridge, MA USA; 13 Vector Institute, Toronto, Ontario Canada; 14 The Ethox Centre and the Wellcome Centre for Ethics and Humanities, Nuffield Department of Population Health, University of Oxford, Oxford, UK; 15 Leeds Teaching Hospitals NHS Trust, Leeds, UK; 16 University of Leeds, Leeds, UK; 17 Department of Clinical Pathology and Department of Clinical and Experimental Medicine, Linköping University, Linköping, Sweden; 18 Center for Medical Image Science and Visualization, Linköping University, Linköping, Sweden; 19 Google Research, Montreal, Quebec Canada; 20 Institute for Health Informatics, University College London, London, UK; 21 Wellcome Trust, London, UK; 22 Patient and Public Involvement and Engagement (PPIE) Group, STANDING Together, Birmingham, UK; 23 Institute of Women’s Health, UCL, London, UK; 24 Oxford University Hospitals NHS Foundation Trust, Oxford, UK; 25 PIONEER, HDR UK Hub in Acute Care, Institute of Inflammation and Ageing, University of Birmingham, Birmingham, UK; 26 National Institute for Health and Care Research, Great Ormond Street Hospital Biomedical Research Centre, London, UK; 27 Great Ormond Street Institute of Child Health, University Hospital London, London, UK; 28 Birmingham Health Partners Centre for Regulatory Science and Innovation, University of Birmingham, Birmingham, UK; 29 Centre for Patient Reported Outcomes Research, Institute of Applied Health Research, College of Medical and Dental Sciences, University of Birmingham, Birmingham, UK; 30 National Institute for Health and Care Research Applied Research Collaboration West Midlands, University of Birmingham, Birmingham, UK; 31 National Institute for Health and Care Research Birmingham–Oxford Blood and Transplant Research Unit in Precision Transplant and Cellular Therapeutics, University of Birmingham, Birmingham, UK; 32 DEMAND Hub, University of Birmingham, Birmingham, UK; 33 UK SPINE, University of Birmingham, Birmingham, UK; 34 National Institute for Health and Care Research Biomedical Research Centre, Moorfields Eye Hospital/University College London, London, UK

**Keywords:** Health policy, Medical ethics, Clinical trial design, Public health

## Abstract

Artificial intelligence as a medical device is increasingly being applied to healthcare for diagnosis, risk stratification and resource allocation. However, a growing body of evidence has highlighted the risk of algorithmic bias, which may perpetuate existing health inequity. This problem arises in part because of systemic inequalities in dataset curation, unequal opportunity to participate in research and inequalities of access. This study aims to explore existing standards, frameworks and best practices for ensuring adequate data diversity in health datasets. Exploring the body of existing literature and expert views is an important step towards the development of consensus-based guidelines. The study comprises two parts: a systematic review of existing standards, frameworks and best practices for healthcare datasets; and a survey and thematic analysis of stakeholder views of bias, health equity and best practices for artificial intelligence as a medical device. We found that the need for dataset diversity was well described in literature, and experts generally favored the development of a robust set of guidelines, but there were mixed views about how these could be implemented practically. The outputs of this study will be used to inform the development of standards for transparency of data diversity in health datasets (the STANDING Together initiative).

## Abstract (web summary)

A systematic review, combined with a stakeholder survey, presents an overview of current practices and recommendations for dataset curation in health, with specific focuses on data diversity and artificial intelligence-based applications.

## Main

Recent years have seen a rapid rise in the development of artificial intelligence (AI) systems for use in healthcare, including those that qualify as a medical device (known as AI as a medical device, AIaMD). This has been enabled by increasing use of electronic health records, accompanied by curation of large-scale health datasets^1^. However, there are credible concerns that many datasets inadequately reflect the diversity of the individuals or groups contained in the population they are intended to represent. This has previously been described as ‘Health Data Poverty’: a phenomenon where individuals or groups who are underrepresented in health datasets are less able to benefit from data-driven innovations developed using these datasets, including AIaMD^2^. There is a growing concern that non-diverse and non-representative data contribute to the creation of biased algorithms, resulting in less accurate performance in certain patient groups. Therefore, it is well-recognized that an essential component of ensuring algorithmic safety is to guarantee that datasets are appropriately diverse and representative of their intended use population^3^.

Data diversity, as measured by equal or relative representation alone, is not enough to achieve equitable outcomes. Even when individuals are represented proportionally in datasets, other forms of bias may be inherently embedded in the representation of those individuals’ data. For example, a dataset may include a proportion of individuals from an ethnic group that is in keeping with national census data (adequate numerical representation), but included individuals from this ethnic group could have a systematically higher likelihood of being misdiagnosed than in the sampled population, meaning the insights derived from this data can remain biased. Although a principal focus of our work is data diversity, we also advocate for a broader view of representativeness in health data, including awareness of the limitations of data collection, data accuracy and ethical concerns around the use of data in minoritized and underserved groups.

**Fig. 1:**
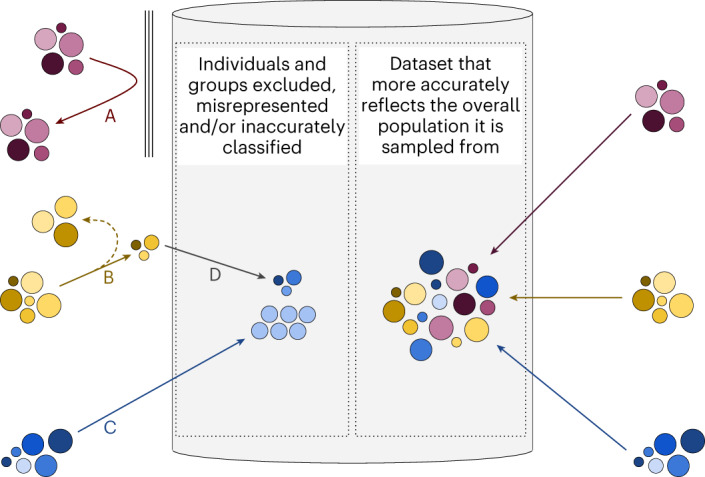
Individuals may be underrepresented in datasets for many reasons. Barriers may be present that prevent data about entire groups of people from being included in the dataset (A). They may include barriers to accessing health or social care (meaning data are not generated), inadvertent or deliberate exclusion by the dataset curators or absence of electronic health records (meaning data are not digitized). Certain individuals may be less likely to enter datasets (B); for example, when individuals choose not to allow their data to be included, when methods for data collection are exclusionary (for instance, forcing a binary choice of ‘male’ or ‘female’ for gender), or when redaction occurs after data collection because of legal or ethical restrictions on data sharing. Data may not be collected in sufficient detail, leading to data loss (C; for instance, capturing age in categorical bands such as 20–29 rather than as a continuous variable). Groups of individuals with distinct personal attributes may be merged into a different group either at the point of data collection or by preprocessing after collection (D; for instance, requiring ethnicity or race to be selected from a small list of choices during data capture or combining ethnicity or race groupings into a larger, aggregate group after data capture).

Reasons for underrepresentation in datasets broadly fall into two categories: factors that cause individuals or groups to be absent from datasets and factors that cause individuals to be incorrectly or inappropriately categorized into groups despite being present (for example, categories of ‘mixed ethnicity’ or ‘other’). Root causes may include structural barriers to receiving healthcare; barriers to the capture or digitization of relevant health data; individual and structural barriers reducing consent for data sharing; data aggregation, redaction or recoding; collecting data with insufficient granularity; and legal or ethical restrictions on data sharing preventing data accessibility (Fig. 1)^2,4,5^. The composition and diversity of teams involved in AIaMD development is also critical—teams should include people from different backgrounds as well as those with lived experience of the use case (for example, patients and the public).

Examples of this lack of diversity in datasets have been previously highlighted in several health areas, including radiology, ophthalmology and dermatology^6–8^. There are further concerns that models may encode biases relating to demographic characteristics even when they are not explicitly trained to do so. This leads to the potential for ‘unknown’ biases reflected in health datasets to become unknowingly and unintentionally embedded in models derived from them^9,10^.

Despite widespread acknowledgement that inclusiveness is a core tenet of ethical AI in healthcare^11–13^, there remains a shortage of guidance on how to apply such principles in the curation, aggregation and use of health data. The issue of producing data relating to healthcare disparities has previously been explored, with recommendations for data-collection practices, but there are novel challenges in the specific context of AI research^14,15^. Generic guidelines exist for the improvement of datasets, including a view to reduce healthcare inequalities by promoting patient voice, accurate variables and data linkage^14^. A commonly raised concern is the reporting of race/ethnicity data, which is variably collected with fragmented and diverse data-collection practices^16^. The US Food and Drug Administration (FDA) asks for demographic information and inclusion criteria for data collection to be provided if available but does not mandate certain levels of representation across demographic groups in datasets used in AIaMD^17^. This is a concern particularly pertinent to the field of AIaMD because of the risk of systemic algorithmic bias if models are trained on biased training datasets. AIaMD algorithms learn patterns in the training data and use this to generate predictions when applied to new data. If the data used for training an algorithm are biased against particular demographic groups, the algorithm is likely to underperform when applied to those groups in the real world. Beyond algorithmic bias, diversity in datasets has wider benefits in improving algorithmic performance. A diverse dataset helps AIaMD models generalize their learnings to new and unseen cases. Without diversity, models may perform well on common cases but struggle with unusual or underrepresented ones.

This Analysis aims to explore existing standards, frameworks and best practices that improve data diversity in health datasets in the context of AIaMD. It comprises two parts: (1) a systematic review of the published literature for existing standards, frameworks and best practices; and (2) a survey of stakeholder views to understand how issues of bias and health equity are tackled at present for AIaMD and how best practices can be promoted in the future. This work is part of the STANDING Together initiative (standards for data diversity, inclusivity and generalizability), a program that seeks to develop consensus-driven standards for health data to promote health equity; further information is available at www.datadiversity.org (ref. ^18^).

## Results

### Systematic review

**Fig. 2:**
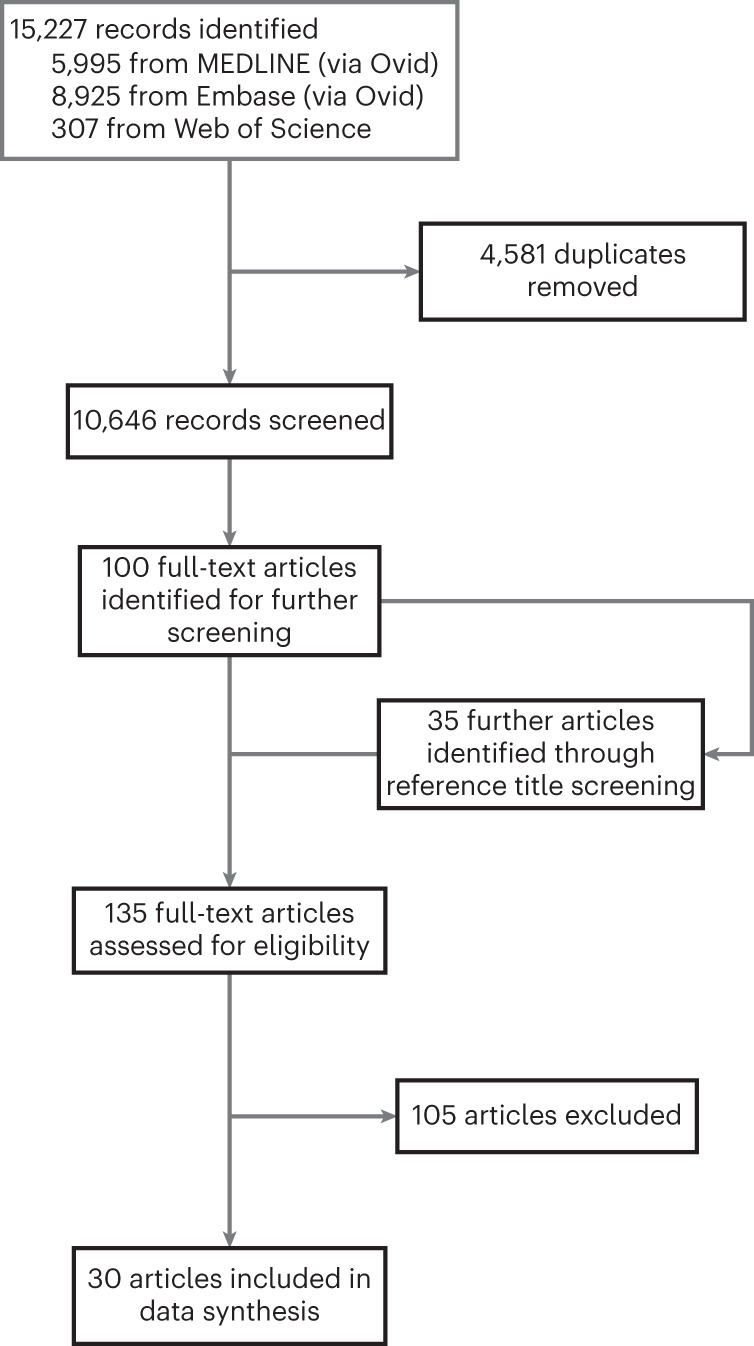
PRISMA flow diagram for systematic review. The breadth of the search strategy meant most of the 10,646 records that were screened were irrelevant and did not meet any of the inclusion criteria, addressing neither health equity nor AIaMD.

Database searches yielded 10,646 unique records, of which 100 remained after title and abstract screening (Fig. 2). Most of the 10,646 records that were screened did not meet the inclusion criteria, addressing neither health equity nor AIaMD. A further 35 records were screened after identification through reference lists. We identified seven arXiv preprints for the analysis through this method. After full-text screening, 30 relevant records were included.

Of these 30 records, 17 were identified from bibliographic databases, 9 from searches of reference lists and 4 from searches of unindexed conference proceedings (Table 1). All 30 were published between July 2015 and February 2022. Of the 30 records, 1 was published in 2015, 1 in 2017 and 28 since 2018, showing acceleration in the rate of academic discussion around this topic. Most records were authored by individuals from the same department in one institution (14 of 30; 47%), with only 4 of 30 (13%) authored by interdisciplinary, international teams^19–22^. Twenty of 30 records (67%) were found in journals, 7 of 30 (23%) were found as preprints and 4 of 30 (13%) were found in conference proceedings. Twenty-seven of 30 (90%) were available open access.

**Table 1:** Characteristics of included articles

Study ID	Title (year)	Article type	Description of record	Access	Type of collaboration
SR1	Heterogeneity/granularity in ethnicity classifications project: the need for refining assessment of health status (2018)^46^	Journal article	Description of how ethnicity is recorded across different EU countries; some collect highly granular data, some allow free text expression, others allow only limited categories	Open access	International collaboration
SR2	Bringing the people back In: contesting benchmark machine learning datasets (2020)^47^	Preprint	Outlines the concept of benchmark datasets as a form of research infrastructure and key factors that may influence a dataset’s value and utility	Open access	National collaboration
SR3	A framework for understanding sources of harm throughout the machine learning life cycle (2019)^24^	Preprint	Maps where biases may cause harm during a ML development pipeline	Open access	Single institution
SR4	Datasheets for datasets (2018)^32^	Preprint	Introduces a ‘Datasheet’ artifact, allowing dataset curators to provide a comprehensive, structured and standardized description of a dataset’s composition and the context in which it has been curated	Open access	National collaboration
SR5	The dataset nutrition label: a framework to drive higher data quality standards (2018)^48^	Preprint	Introduces a ‘Nutrition label’ artifact, allowing dataset curators to provide a structured, standardized summary of a dataset’s composition	Open access	National collaboration
SR6	Ensuring that biomedical AI benefits diverse populations (2021)^11^	Journal article	Highlights how AI development can cause biases and health disparity. Also indicates both short-term and longer-term solutions to mitigate some of these factors	Open access	Single institution
SR7	How to design AI for social good: seven essential factors (2020)^49^	Journal article	Identifies and explains seven essential ethical factors to consider when developing AI for social good. Each factor is followed by a recommendation for developers who are seeking to develop AI that promotes social good	Open access	National collaboration
SR8	Identifying ethical considerations for machine learning healthcare applications (2020)^50^	Journal article	Framework linking the ML development pipeline to evaluation and oversight of these technologies, highlighting where along this joint pathway ethical considerations and value-based issues may arise	Closed access	National collaboration
SR9	Indigenous and tribal peoples data governance in health research: a systematic review (2021)^51^	Journal article	Systematic review of data governance frameworks, processes, policies and practices for indigenous and tribal peoples	Open access	Single institution
SR10	MINIMAR (MINimum Information for Medical AI Reporting): developing reporting standards for artificial intelligence in health care (2020)^52^	Journal article	Minimum reporting standards for studies of medical AI, relating to the study population and setting, patient demographic characteristics, model architecture and model evaluation	Open access	Single institution
SR11	Predictably unequal: understanding and addressing concerns that algorithmic clinical prediction may increase health disparities (2020)^53^	Journal article	Ethical discussion about the differences between algorithmic fairness and bias and a summary of different definitions of fairness	Open access	Single institution
SR12	The reporting of race and ethnicity in medical and science journals: comments invited (2021)^26^	Journal article	Guidance for reporting ethnicity and race in research articles specifically for JAMA Network journals	Open access	Single institution
SR13	Ethical limitations of algorithmic fairness solutions in health care machine learning (2020)^54^	Journal article	Commentary on how framing algorithmic fairness as entirely a technical problem can contribute to or cause health inequity unless social factors are also considered	Open access	National collaboration
SR14	Missed policy opportunities to advance health equity by recording demographic data in electronic health records (2015)^55^	Journal article	Description of how different US bodies and organizations take different approaches to collecting demographic data, including using different categories, which limits crosslinking between data sources	Closed access	Single institution
SR15	Clinical collabsheets: 53 questions to guide a clinical collaboration (2020)^22^	Conference proceedings	A guide to collaborating between clinicians and computer scientists to develop models in interdisciplinary teams across eight development stages	Open access	Multidisciplinary international collaboration
SR16	Ethical machine learning in healthcare (2021)^5^	Journal article	Overview of the five key stages in the healthcare ML model development pipeline, overlaying points at which ethical issues may arise	Open access	International collaboration
SR17	Addressing health disparities in the Food and Drug Administration’s artificial intelligence and machine learning regulatory framework (2020)^23^	Journal article	Commentary about how health disparities might be considered by the FDA software as a medical-device regulatory framework, through integration of premarket review and good ML practices and postmarket real-world performance monitoring	Open access	Single institution
SR18	Model cards for model reporting (2018)^56^	Preprint	Introduces a ‘Model card’ artifact, encouraging transparent reporting of ML model performance characteristics	Open access	National collaboration
SR19	Canada protocol: an ethical checklist for the use of artificial intelligence in suicide prevention and mental health (2019)^57^	Preprint	An ethical checklist for the use of AI in mental health and suicide prevention, validated by two-round Delphi consultation. Note that a version of this record was subsequently published closed access in a journal^57^	Open access	Single institution
SR20	Aequitas: a bias and fairness audit toolkit. (2018)^58^	Preprint	An open-source bias audit toolkit to allow ML developers, analysts and policymakers to assess AI systems for biased outputs	Open access	Single institution
SR21	AI-assisted decision-making in healthcare: the application of an ethics framework for big data in health and research (2019)^25^	Journal article	A discussion of key ethical issues involved with AI implementation in healthcare, with specific case study examples	Open access	National collaboration
SR22	An ethics framework for big data in health and research (2019)^59^	Journal article	A framework of values underpinning ethical design of AI in healthcare, developed by a working group with expert feedback	Open access	International collaboration
SR23	Artificial intelligence for genomic medicine—a policy analysis (2020)^60^	Conference proceedings	Practical recommendations for policymakers in the field of AI and genomic medicine, exploring the drivers behind the use of AI in genomics, current applications and limitations and challenges	Open access	Single institution
SR24	Big data science: opportunities and challenges to address minority health and health disparities in the 21st century (2017)^61^	Journal article	A discussion of how big data science can be used to address minority health issues and actively reduce health disparities by changing the types and mechanisms of electronic health-data capture and enabling studies into health disparities. Also provides a series of recommendations to achieve these aims	Open access	National collaboration
SR25	Ensuring fairness in machine learning to advance health equity (2018)^62^	Journal article	Describes how health disparities can be worsened by model design, data biases and interpretation by patients and clinicians. Recommends that proactive distributive justice be incorporated into models to ensure equality in patient outcomes, resource allocation and model performance	Open access	Single institution
SR26	Machine learning and artificial intelligence research for patient benefit: 20 critical questions on transparency, replicability, ethics and effectiveness (2020)^19^	Journal article	Framework for interdisciplinary groups researching, generating or implementing ML models to determine a model’s potential to benefit patients. Focuses on transparency, replicability, ethics and effectiveness	Open access	Multidisciplinary international collaboration
SR27	Do no harm: a roadmap for responsible machine learning for health care (2019)^21^	Journal article	A set of principles promoting practices that enable acceleration of translation of ethical and effective ML models in healthcare, spanning problem selection, development, ethical considerations, evaluation and reporting, deployment and postmarket considerations	Open access	Multidisciplinary international collaboration
SR28	Addressing fairness, bias and appropriate use of artificial intelligence and machine learning in global health (2021)^63^	Journal article	A framework for those deploying ML algorithms in low- and middle-income countries, focusing on determining whether a model is appropriately matched to the local context and target population, identifying biased performance and considering implications for fairness	Closed access	Single institution
SR29	Artificial intelligence, bias and clinical safety (2019)^20^	Conference proceedings	Discussion of potential medical AI errors and biases and presentation of quality-control questions enabling critical appraisal of medical AI research and highlighting potential pitfalls for future researchers	Open access	Multidisciplinary international collaboration
SR30	Healthsheet: development of a transparency artifact for health datasets (2022)^33^	Journal article	Introduces a ‘Healthsheet’ artifact, allowing healthcare dataset curators to provide a comprehensive, structured and standardized description of a dataset’s composition and the context in which it has been curated. Related to the ‘Datasheet’ artifact, but adapted for healthcare datasets	Open access	Single institution

Results of literature search, including sources found through journal database searches, preprint servers and reference lists.

### Themes from the literature review

Data were extracted from all 30 records to derive key themes (Extended Data Table 1). Transparency around data-collection practices was a major theme (*n* = 13), with particular focus on the need for clarity about how data were sampled and for what purpose, how demographic categories were assigned and details of any preprocessing of data. Some articles highlighted the importance of reporting existing health inequalities affecting those included in a dataset, allowing data users to take steps to avoid exacerbating these^23,24^. The motivation for collecting the data and whether informed consent was obtained from participants was discussed by 11 articles. Furthermore, as legal requirements vary across jurisdictions, there was little consensus about whether or how consent should be obtained from subjects in datasets. Finally, two records discussed how data quality can be improved by involving clinical experts when developing the data-collection strategy^5,25^.

Missing data were discussed in 10 of 30 records (Extended Data Table 2). Transparency about the causes, extent and consequences of missing data is encouraged, as is transparency about any steps taken to address them. It is understood that increased information about the amount of missing data in a dataset will promote transparency and considerations about appropriateness of use, given that usefulness and generalizability of AI and machine learning (ML) models are impaired by missing data^19^. Research into missing data also revealed a recommendation that aggregation of demographic groups or variables into a smaller number of groups should be reported, which would similarly help researchers understand the limitations of the dataset^26^.

Data labeling was addressed by 8 of 30 records (Extended Data Table 3). Transparency was again an overarching theme: articles discuss the need to report how labeling was performed—particularly whether labels represent ground truths and whether there are known or potential biases in labels, such as if they were reported by humans (and therefore subject to inter-reporter variability). One article encouraged analysis of statistical relationships between labels and demographic factors so that potential confounders can be identified and controlled for during model development^5^.

A recurring theme was the identification of groups at risk of harm. These groups were variably described by authors as “vulnerable”, “minority”, “minoritized”, “underserved”, “marginalized” and “protected”. The cross-cutting theme was that these are groups considered to be more susceptible to physical, social or economic harm. The issue of certain groups being at greater risk of vulnerability was discussed in 24 of 30 records (Extended Data Table 4). However, there was little consensus about how biases should be addressed or which groups are most at risk. Suggested approaches to identify and reduce bias and harms for demographic subgroups included predefining groups suspected to be at risk, targeting data collection and model development to benefit these groups in particular, ensuring that representatives from at-risk groups are involved with model development (including as experts in a development team, such as developers, programmers and analysts), testing data for confounders rather than automatically including all features in training data and testing model performance in minoritized subgroups. Attributes specified as being particularly at risk of harm with underrepresentation include ethnicity, race, pregnancy status, age, nationality, gender, sex, socioeconomic status, religion, indigenous and tribal community membership, disability status, sexual orientation, preferred language, Fitzpatrick skin type, health status, education, employment status, geographical location and marital status.

### Stakeholder survey

Whereas the systematic review provided an oversight of current best-practice principles for health datasets in AI, the stakeholder survey provided insights into how principles could be operationalized and by whom. Twenty participants completed the scoping survey. Of these participants, ten (50%) reported their sex as female, nine (45%) reported their sex as male, and one (5%) did not provide this information. Eighteen participants (90%) reported that their gender identity was the same as the sex registered at birth, one participant (5%) reported that their gender identity was different than their sex registered at birth and one participant (5%) did not provide this information. Four main themes and 17 subthemes were identified (Table 2).

**Table 2:** Themes arising from the stakeholder survey

Themes	Subthemes
Theme 1—The role of demographic data	1.1 Current use of demographic data
	1.2 Representativeness of the data
	1.3 Determining importance of race/ethnicity data
	1.4 Challenges of ensuring diversity in datasets
	1.5 Solutions and aspirations to overcome challenges
Theme 2—Data diversity	2.1 Conceptual definition
	2.2 Components of diversity
	2.3 Operationalizing diversity
Theme 3—The use of metrics	3.1 Externally validated or self-report
	3.2 Types of measures
	3.3 Rating diversity
Theme 4—Standards	4.1 Are standards important and needed?
	4.2 Existing standards
	4.3 Recommendations for adoption of standards
	4.4 Recommendations and barriers to adherence
	4.5 Responsibility of adherence
	4.6 Consequences of mandating standards

The first theme was ‘the role of demographic data’. Stakeholders used demographic data in several ways to assess the safety and efficacy of AIaMDs across different subgroup populations. Ensuring that representative data are used to train and validate AIaMDs for the population in which they are to be deployed was felt to be most important. Ways of demonstrating representativeness included describing the intended use and users of the AIaMD, identifying subgroups of interest up front and being transparent about poor performance. Race and ethnicity data were seen as an important means to explore known and unknown biases potentially leading to health inequalities.

*“Models must be able to ‘work’ for those belonging to racialised minority groups, and clinicians/researchers/developers must go through stringent governance measures to ensure inequalities, racism and other forms of discrimination are not exacerbated by use of medical AI models.”*

Challenges of ensuring diversity in datasets included issues of lack of health data in certain populations (health data poverty), lack of standardization across attribute categories, difficulty in harmonizing several methods of data capture and data-governance restrictions. Other factors relating to the development pathway included poorly defined use cases, a lack of relevant stakeholder input, difficulty in accessing suitable datasets and existing gaps in current evidence about underserved populations at risk of harm linked to health outcomes. Stakeholders used various solutions to derive missing demographic data, including statistical techniques such as imputation, Bayesian geocoding and linking across several datasets. Stakeholders suggested they would like to see policy changes, standards of best practice with a statement of scope up front describing data diversity and regulatory authorities providing clarity about specific requirements according to the intended use, as well as toolkits to tackle health data poverty issues.

*“Assist various bodies in addressing data poverty (I see this as the core problem that needs solving).”*

*“Using linked data records and collating ethnicity information from different parts of the healthcare record can improve ethnicity data capture.”*

*“Multiple imputations can be helpful for some variables but is sub-optimal. There are weak surrogates/indirect methods of increasing populating ethnicity variables.”*

The second theme was ‘data diversity’. The definition of dataset diversity was generally considered a universal concept whose application should be context specific depending on the research question or intended use population. The scope of data diversity should be broad and include race, ethnicity, age, gender, sex, socioeconomic status, clinically relevant disease populations, neurodiversity, disability, language barriers and educational level. It was felt that an ideal dataset should represent a global population that is diverse enough to enable a range of problems to be explored with adequate statistical power. Race and ethnicity were seen as dataset attributes that ‘must be included’. Barriers identified included lack of standardization across the globe, low statistical power in underrepresented groups/rare events and a lack of knowledge about intended use populations/subgroups and their health outcomes. Although race and ethnicity are nearly universally acknowledged as important, there is considerable lack of precision and understanding of the terms in science more broadly^27^.

*“Diversity as a concept is universal, but its application is contextual.”*

*“Diversity in health datasets should be used more broadly than simply protected characteristics such as sex and ethnicity.”*

The third theme was ‘the use of metrics’. In principle, most respondents were in favor of some type of metric to measure diversity but unsure about how this could be operationalized. There was a definite lack of consensus about the concept of introducing a rating for the diversity of a dataset, with considerable concerns about how a rating of diversity could be implemented. When metrics were considered, stakeholders felt they would have greater value if externally validated rather than solely reported by the dataset’s curators. An alternative approach was that dataset curators could be validated as ‘safe providers’, meaning curators and/or their organizations would demonstrate adherence to standards across all datasets they produce. Other metrics put forward included level of inclusion, completeness and/or missingness of demographic data and distribution of the data. Interestingly, participants also proposed metrics that were related indirectly to the data itself but rather to the model derived. For example, participants discussed measures of model performance (such as systematic error rates across subgroups) that could result from biases in the data. However, respondents anticipated many challenges, such as knowing up front for which subpopulations poor performance should be specifically tested, a lack of established methods for evaluating performance and comparability of variables across different datasets.

*“I am not sure this could be distilled into a simple set of metrics but rather see a minimum requirement of descriptive information re: a dataset as an option that is proportion of different ethnicities, age groups, gender.”*

*“I don’t think datasets should be ‘rated’ on demographic diversity personally. To me, this borders on saying some are ‘better’ than others but it is context-specific and depends on the planned setting of deployment.”*

The fourth theme was ‘standards’. There was consensus about the importance of standards to enable risks and harms to be identified, improve the quality of the datasets, address bias and provide accountability. However, respondents cautioned against imposing strict diversity requirements, as doing so might risk products being withdrawn for already-marginalized populations, as well as unethical data-gathering practices^28^. Recommendations for adoption included making the standards part of the procurement, funding or product-approval process; making them part of the publishing pathway; or implementing an accreditation pathway for organizations to demonstrate compliance. However, there was no consensus about which organizations should be responsible for mandating compliance.

*“It’s great that the issue has been recognised, but more needs to be done to change things, for example by having a standard for researchers to consider and apply when designing their studies or curating new datasets, and developing methods to ensure people are held accountable.”*

Regarding whether such standards should be mandated, some respondents suggested that mandating the standards through regulators, journal editors or commissioners was an acceptable approach, provided diversity of the populations in the dataset was the requirement. If diversity across populations related to performance of the model was to be mandated for compliance, this approach risked setting a level that could not be achieved by creating a problem with enforcement and increasing costs. The consequences might include slowing or even stifling research and innovation because of data poverty issues. Some felt that a softer approach was needed, such as defining best practice with a set of data diversity standards and encouraging adoption and self-reporting. Whichever enforcement mechanism is implemented, practical tools can be developed to improve uptake, such as providing guidance on how to adhere to the standards, making validation datasets available, engagement with stakeholders, providing incentives and removing barriers. The greatest barriers to adherence identified were resources such as time and cost.

*“Funders at the proposal stage, journals at the publication stage and in parallel the regulatory system.”*

*“If you propose a body for checking compliance more formally, you will need to fund them as well. However, if the stakeholders who are required to comply with the standards feel there is just one more thing being added to their increasing requirements, you may risk facing a rebellion unless this work is adequately compensated. I suggest soft standards rather than formal checks.”*

## Discussion

This study used two methods: a systematic review and a scoping survey of expert stakeholders. The systematic review identified a range of recommendations related to data diversity, with the scoping survey supplementing it by exploring practical considerations relating to their implementation. To our knowledge, this is the first systematic synthesis of existing standards, frameworks, guidelines and best practices for the use of health datasets in the context of AIaMD. We found a clear consensus that there is a need for diversity in datasets and that issues of algorithmic bias may prevail where this is absent. The increasing resources directed towards creating large-scale health datasets has not been accompanied by equivalent efforts to ensure that they are adequately representative and diverse. Even when guidance recognizes the importance of data diversity, it is high-level and not easily operationalized.

The systematic review provided insight into existing guidelines for data collection, handling missing data and labeling data. A key theme found through the systematic review was a need for transparency in how datasets are prepared, including who is included or excluded from the dataset, how missing data are handled and how data are labeled. Greater transparency in these areas allows better understanding of the context and limitations of a dataset, which in turn provides a guide to the potential limitations of any inferences or innovations derived from that dataset. For example, if a dataset excludes certain groups, this information should be evident to potential users of the dataset and should be reported alongside insights derived from that dataset to provide context and the likely scope of application.

With regard to personal attributes, among all included articles, sex, age, race and ethnicity were most commonly cited as attributes that could associate a group with risk of harm or disadvantage; however, there was a notable lack of literature addressing how these concerns could be addressed for these groups. Less commonly, records cited pregnancy, income, marital status and a range of other attributes that may require further consideration. There was notable overlap between attributes identified as often lacking diversity and those that are specifically protected by various jurisdictions, such as in the UK Equality Act 2010, the European Union Charter of Fundamental Rights and the United Nations^29–31^. The importance of collecting demographic attribute information is not necessarily for it to be embedded as an input variable or predictor in ML models (which may perpetuate harm along these axes) but importantly, to ensure that it is collected for auditing model performance across disaggregated subgroups as a method of bias discovery. In our literature review, ‘Healthsheet’ and ‘Datasheets for Datasets’ were the most comprehensive guides to data documentation, with elements in both relating to diversity^32,33^.

There was a high degree of concordance between the recommendations gathered from the systematic review and those obtained through our survey. However, the scoping survey highlighted the potential difficulties and lack of pragmatic guidance about how such guidelines can be implemented practically as well as who should be responsible for overseeing their implementation. This indicates that the issues of interest are well-recognized and conceptually understood; however, there is a clear need to focus on operationalizing existing knowledge. The scoping survey additionally identified barriers and potential enablers to creating standards and best practices that are ready for translation into the real world. It highlighted that although standards would be beneficial, there must be some means of implementing them, which could include embedding them into a product-approval process or making them prerequisites for eligibility for funding, health technology appraisal or procurement. Standards could also be implemented by externally provided accreditation, which may be seen favorably by funders, regulatory bodies and research boards. Another option includes voluntary self-reporting of standards with minimal external oversight.

It was also highlighted that it is not clear which agencies could mandate or suggest completion of such standards. Journal editors, regulators, health policy organizations and funders were among the suggested agencies. In health research, the Enhancing the Quality and Transparency of Health Research (EQUATOR) network is well-recognized as a resource for gold-standard guidance for study reporting, endorsing widely used guidelines including Consolidated Standards of Reporting Trials 2010 guidelines for randomized controlled trials. Although the network focuses on reporting and transparency of research studies, a similar structure may be considered for datasets. The role of regulatory agencies in mandating the data diversity used in AIaMD is unclear, as their remit is at present limited to ensuring that datasets reflect the intended use population. Although respondents acknowledged the lack of evidence on this topic, a counterargument to heavy-handed enforcement was also raised from the stakeholder survey, with the perception that enforcing higher expectations for datasets may stifle innovation, impede health improvement and possibly exacerbate inequalities as a result. Overall, the findings of the survey indicated strong support for the development of standards but ambiguity as to their implementation.

This study involved a comprehensive review of academic literature reporting recommendations for dataset use, but it did not extend to reviewing gray literature, including governmental reports. However, we have noted that the issue of algorithmic bias has been formally recognized by the UK Medicines and Healthcare products Regulatory Agency, UK government, the UK Information Commissioner’s Office, the US FDA and the European Parliament^34–39^. “Ensuring inclusiveness and equity” is one of six principles for AI development prescribed by the World Health Organization, referring to “age, sex, gender, income, race, ethnicity, sexual orientation, ability or other characteristics protected under human rights codes”^13^. The same WHO report states, “No technology, AI or otherwise, should sustain or worsen existing forms of bias and discrimination”^13^.

The search was limited to records published since 2015. This is a limitation to the study, but it is noted that the results exhibited an exponential increase in the number of relevant records published since 2018. Only one relevant record was published in 2015. It would be expected that any notable pre-2015 papers should have been revealed in reference-list screening, but none were. Accordingly, setting an earlier start date for screening would have been unlikely to identify any more relevant articles and would have substantially increased the number of papers it was necessary to screen. Data collection was performed by two independent reviewers for 11 of the records. Data for the remaining articles were extracted by a single reviewer, who extracted data from all papers included in the analysis for consistency of voice in the extracted summaries. Although data collection by a single author is a limitation, use of a standardized data-extraction sheet partially mitigates it.

This study has focused on factors that encourage dataset diversification as a lever to address health data poverty. Underrepresentation of minority groups in datasets is well-recognized as an important driver of algorithmic bias, but other mechanisms can be applied downstream to mitigate its effects. This includes practicing model diversity: for example, producing several models and combining the outputs with ensemble learning to diversify the parameters considered by the model and reduce the risk of overfitting to an unbalanced data sample. Synthetic data have also been recently proposed as a method of selectively generating data for marginalized populations to rebalance datasets; however, this approach has limitations in that it effectively oversamples from a small group without truly gaining diversity^40–42^. It should be emphasized that these (and other) methods are an active area of research with a need for empirical evidence to prove their applicability. Similarly, this review does not extend into other ethical implications of the use of ML in healthcare, although this topic has been extensively studied previously and includes other issues of privacy, trust and accountability^43^.

We took a broad approach to the recruitment of different types of stakeholders in the scoping survey and therefore did not apply a formal sampling framework. As a result, we cannot be sure the voices represented are consistent across all stakeholders. This survey was intended as a scoping exercise and not meant to be an exhaustive qualitative study. Future planned work as part of the STANDING Together initiative involves input from a wider gamut of stakeholders including patient and public partners (two of whom are coauthors of this study).

The issue of algorithmic bias is well described in medical literature, with dataset insufficiency a key driver. Although principles such as Findable, Accessible, Interoperable, and Reusable (FAIR) seek to improve data availability and use more generally, there is a paucity of data for certain groups and lack of diversity in existing datasets^44^. This Analysis has highlighted the importance of curating and aggregating health data to promote diversity, inclusivity and equity as well as the lack of guidelines available to facilitate doing so. Although reporting guidelines exist for randomized controlled trials using AI, they focus on the reporting of study results rather than the design and use of datasets^45^. Future avenues of research may seek to produce clear guidelines for the development and use of datasets, revolving around the need for diversity and inclusion of marginalized populations and improving data interoperability by means of common data models and standards such as Observational Medical Outcomes Partnership (OMOP) and Fast Healthcare Interoperability Resources (FHIR). Specific guidance about new development practices (including use of synthetic data, federated learning and foundation models) is also needed.

Transparent documentation around diversity and appropriateness of datasets used in AIaMD development will help commissioners, clinicians and health systems determine the risk of bias so they can make informed decisions around whether to deploy corresponding algorithms for their population. The STANDING Together project (https://www.datadiversity.org/) is one such endeavor. Building on the outputs of this systematic review and stakeholder survey, STANDING Together is developing consensus-derived standards coauthored by an international, interdisciplinary team that reinforce ethics and inclusivity in the documentation and use of healthcare datasets, allowing developers to ensure that AIaMD works for everyone^18^. The findings described in this literature review and stakeholder survey will directly inform the proposed items for STANDING Together, and the methods describing their translation into specific, actionable recommendations will be outlined in a subsequent paper. The recommendations will undergo a multistakeholder, three-staged Delphi study, and the resulting standards will be available in late 2023.

## Methods

This research was conducted in compliance with all relevant ethical regulations, including informed consent from all participants. Ethical approval was granted by the University of Birmingham’s Science, Technology, Engineering and Mathematics Ethical Review Committee (ERN_21-1831).

### Systematic review

We searched for records describing existing standards, frameworks and best practices for ensuring data diversity in health datasets in the context of AIaMD. An informatician was consulted for the development of the search strategy (Systematic Review Search Strategy), and the searches were conducted on 10 October 2021 on MEDLINE (Ovid), Embase (Ovid) and Web of Science. Results were limited to publications since 2015 in the English language. E-publications ahead of print, in-process publications, in-data-review and other non-indexed citations were included through the MEDLINE search. Deduplication was carried out in EndNote 20 (Clarivate, 2013) and screening in Covidence (https://www.covidence.org/; Veritas Health Innovation, 2022). It was recognized that relevant results may exist as preprints that may not be covered by traditional systematic review searches. As preprint databases are not typically covered by searchable databases, nor are their websites conducive to systematic searches, the burden of the workload to replicate our strategy on the medRxiv and arXiv engines would have been unmanageable. MedRxiv is indexed by Embase, but arXiv is not indexed by any of the databases used. To mitigate this limitation, we conducted reference-list screening for all 100 articles included for full-text screening. Reference lists of included records were searched to identify relevant preprints (including arXiv and medRxiv) or other potentially relevant records. Further searches were conducted of the past five years’ worth of archives of relevant conference proceedings for ML and AI in healthcare: ‘Machine Learning for Health’ (https://ml4health.github.io/2022/), ‘Machine Learning for Healthcare’ (https://www.mlforhc.org) and ‘Conference on Health, Inference, and Learning’ (https://www.chilconference.org/). This scoping review was conducted following the recommendations of the Preferred Reporting Items for Systematic Reviews and Meta-Analyses (PRISMA) statement, and a PRISMA flow chart was also created (Fig. 2)^64^. Abstract screening was performed by two authors independently (A.A., J.A. or X.L.). Non-consensus was resolved by discussion and involvement of a third reviewer if necessary. Before full-text screening, 10% of the records were full-text screened independently by two reviewers as part of a pilot. Once a high degree of concordance was reached between two independent reviewers, the remaining records were assessed by a single reviewer. Studies were included if they presented standards, frameworks or guidance for AI or health data about issues intersecting AI and bias, fairness, health equity and representation, and coding/categorization of minoritized and marginalized groups. Exclusion criteria were guidance not related to health data, guidance relating to technical and infrastructural aspects of health dataset curation only, guidance relating to privacy and governance and cybersecurity only.

For each record, a single reviewer extracted data using a predefined data-extraction sheet. Bibliometric information about each record was extracted, including publication date, number of citations and details of the authorship team (in terms of single/several institutions, geographies and disciplinary background). If specific vulnerable or minority patient groups were discussed in the record, they were also recorded separately. When it was reported, we extracted information about the methodology that led to the construction of the recommendations, including descriptions of any literature review and stakeholder involvement.

### Stakeholder survey

We approached 45 participants representing individuals who work in health-data research and/or AIaMD, including dataset curators, academics, clinicians and medical-device regulators. Participants were identified as authors of relevant publications and through consultation with the STANDING Together working group on the basis of expertise and previous work in healthy inequity, medical datasets and AIaMD. Respondents were invited by email to participate in the survey using Qualtrics (Qualtrics XM, 2018; https://www.qualtrics.com).

The survey consisted of 14 free-text questions (Supplementary File 5) exploring how issues of bias and risk of health inequity are at present tackled for AIaMD and how best-practice recommendations could be operationalized. For data analysis, we took an inductive thematic analysis approach that was exploratory and descriptive in nature^65^. One author (J.P.) conducted the analysis and used several iterations of the responses to the survey questions to refine the initial list of codes and create a codebook using NVivo (NVivo release 1.0, March 2020, QSR International; https://lumivero.com/products/nvivo/). Two authors (E.L. and X.L.) reviewed the data extracts and relevant codes independently, and then all three of the above authors discussed and agreed on the final codebook.

### Statistics and reproducibility

Data relating to the effect of articles included in our systematic review (including journal impact factor, citation count and altmetric data) were obtained but not included in the analysis because these data were not necessary to extract themes from the included articles. No statistical method was used to predetermine the sample size for the stakeholder survey. Blinding and randomization are not applicable to non-interventional studies.

### Systematic review search strategy

#### MEDLINE search strategy

Ovid MEDLINE(R) and Epub Ahead of Print, In-Process, In-Data-Review & Other Non-Indexed Citations, Daily and Versions(R) <1946 to October 08, 2021>

1. cultural diversity/ 12175
2. (ethic* or divers* or fairness or fair or bias or biased or pluralism* or multicultural*).mp. [mp=title, abstract, original title, name of substance word, subject heading word, floating subheading word, keyword heading word, organism supplementary concept word, protocol supplementary concept word, rare disease supplementary concept word, unique identifier, synonyms] 1124521
3. 1 or 2 1124521
4. exp Artificial Intelligence/ 124778
5. (Artificial intelligence or AI or Natural Language processing or NLP or Machine learning or Support Vector Machine* or neural network* or deep learning).mp. [mp=title, abstract, original title, name of substance word, subject heading word, floating sub-heading word, keyword heading word, organism supplementary concept word, protocol supplementary concept word, rare disease supplementary concept word, unique identifier, synonyms] 196657
6. ((health or patient or medical) adj2 (data* or record*)).mp. [mp=title, abstract, original title, name of substance word, subject heading word, floating sub-heading word, keyword heading word, organism supplementary concept word, protocol supplementary concept word, rare disease supplementary concept word, unique identifier, synonyms] 324576
7. exp Medical Records/ 152836
8. 4 or 5 or 6 or 7 587250
9. guideline/ 16447
10. (standard* or guidance or guideline* or framework* or policy or policies or governance).mp. [mp=title, abstract, original title, name of substance word, subject heading word, floating sub-heading word, keyword heading word, organism supplementary concept word, protocol supplementary concept word, rare disease supplementary concept word, unique identifier, synonyms] 3063858
11. 9 or 10 3063858
12. 3 and 8 and 11 9897
13. limit 12 to yr=“2015 -Current” 6089
14. limit 13 to lg=“english” 5995

https://ovidsp.ovid.com/athens/ovidweb.cgi?T=JS&NEWS=N&PAGE=main&SHAREDSEARCHID=5AewcQNdzUJq8nhWGWvzW9upsUXpJwx4ATn6wMF7oYIff436XcLpXSvsrKDfq9BH1

#### Embase search strategy

Embase <1996 to 2021 Week 40>

1. cultural diversity/ 2415
2. (ethic* or divers* or fairness or fair or bias or biased or pluralism* or multicultural*).mp. [mp=title, abstract, heading word, drug trade name, original title, device manufacturer, drug manufacturer, device trade name, keyword heading word, floating subheading word, candidate term word] 1235079
3. 1 or 2 1235079
4. exp Artificial Intelligence/ 51842
5. (Artificial intelligence or AI or Natural Language processing or NLP or Machine Learning or Support Vector Machine* or neural network* or deep learning).mp. [mp=title, abstract, heading word, drug trade name, original title, device manufacturer, drug manufacturer, device trade name, keyword heading word, floating subheading word, candidate term word] 237951
6. ((health or patient or medical) adj2 (data* or record*)).mp. [mp=title, abstract, heading word, drug trade name, original title, device manufacturer, drug manufacturer, device trade name, keyword heading word, floating subheading word, candidate term word] 582323
7. exp medical record/ 255321
8. 4 or 5 or 6 or 7 822925
9. (standard* or guidance or guideline* or framework* or policy or policies or governance).mp. [mp=title, abstract, heading word, drug trade name, original title, device manufacturer, drug manufacturer, device trade name, keyword heading word, floating subheading word, candidate term word] 3535573
10. 3 and 8 and 9 13849
11. limit 10 to yr=“2015 -Current” 8994
12. limit 11 to lg=“english” 8925

https://ovidsp.ovid.com/athens/ovidweb.cgi?T=JS&NEWS=N&PAGE=main&SHAREDSEARCHID=5R3d9uJan3qknMl4p9Tr8MZjsl3nZURHqnXj2n1bjSF8UFTCBrrkGOdGy8x5CCWQ0

#### Web of Science strategy

((((((((TS=(“ethic*”)) OR TS=(“divers*”)) OR TS=(“fairness”)) OR TS=(“fair”)) OR TS=(“bias”)) OR TS=(“biased”)) OR TS=(“pluralism*”)) OR TS=(“multicultural*”)) AND ((((((((TS=(“Artificial Intelligence”)) OR TS=(“AI”)) OR TS=(“Natural Language processing”)) OR TS=(“NLP*”)) OR TS=(“Machine Learning”)) OR TS=(“Support Vector Machine*”)) OR TS=(“neural network*”)) OR TS=(“deep learning”)) AND TS=((“health” OR “patient” OR “medical”) NEAR/2 (“data*” OR “record*”)) AND (((((((TS=(“standard*”)) OR TS=(“guidance”)) OR TS=(“guideline*”)) OR TS=(“framework*”)) OR TS=(“policy”)) OR TS=(“policies”)) OR TS=(“governance”))

Refined to results from 2015

Refined to results in English language

307 results

https://www.webofscience.com/wos/woscc/summary/35bc8104-7d84-4a19-a5a8-9fb366bea050-0c3ff05a/relevance/1

### Reporting summary

Further information on the research design is available in the Nature Portfolio Reporting Summary linked to this article.

## Online content

Any methods, additional references, Nature Portfolio reporting summaries, source data, extended data, supplementary information, acknowledgements, peer review information; details of author contributions and competing interests; and statements of data and code availability are available at 10.1038/s41591-023-02608-w.

### Supplementary information

Supplementary Information Scoping survey questions.

Reporting Summary

## Figures and Tables

**Extended Data Table 1:**
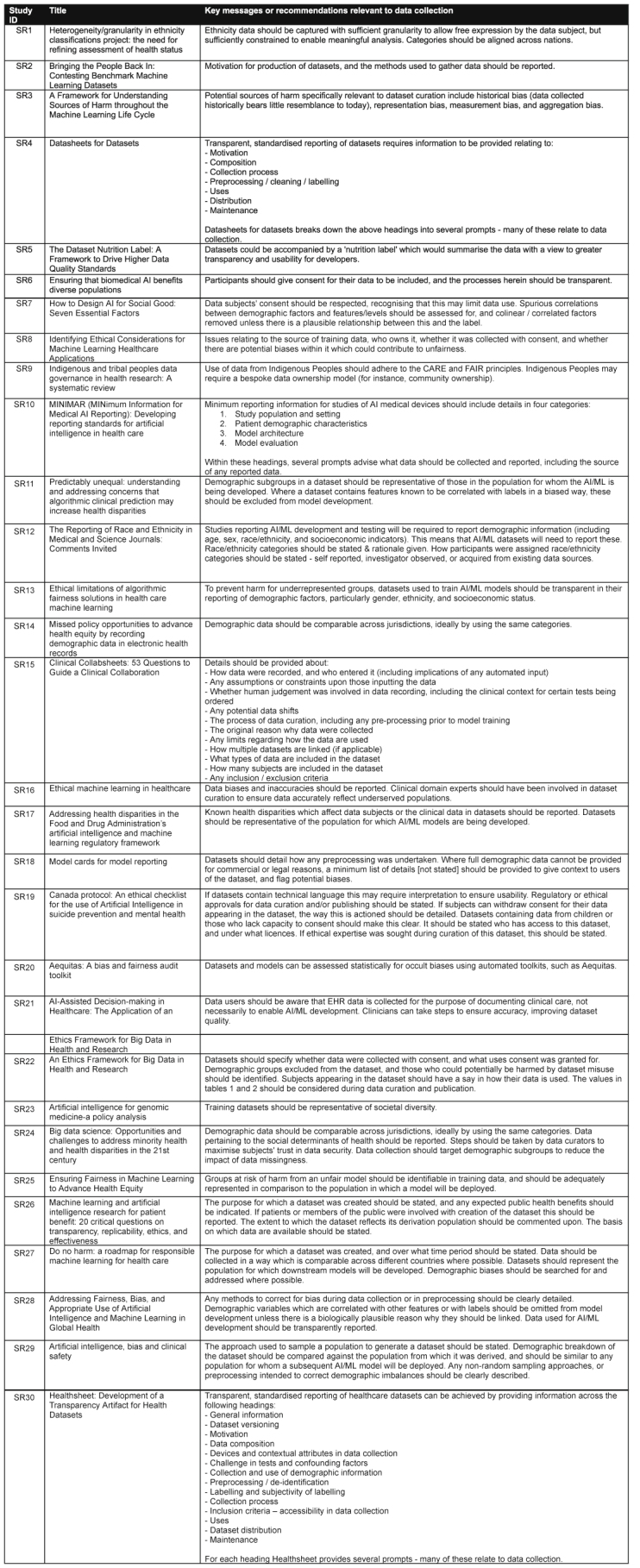
Key messages or recommendations relevant to data collection

Key messages or recommendations extracted from each source relating to data collection and how this may be a cause of bias.

**Extended Data Table 2:**
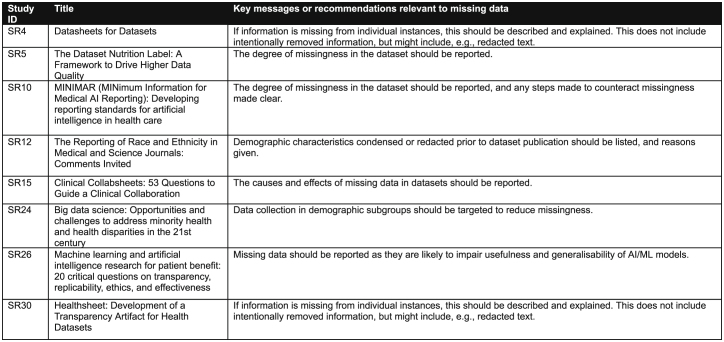
Key messages or recommendations relevant to missing data

Key messages or recommendations extracted from each source relating to missing data (and any steps taken to prevent or correct missing data) and how this may be a cause of bias.

**Extended Data Table 3:**
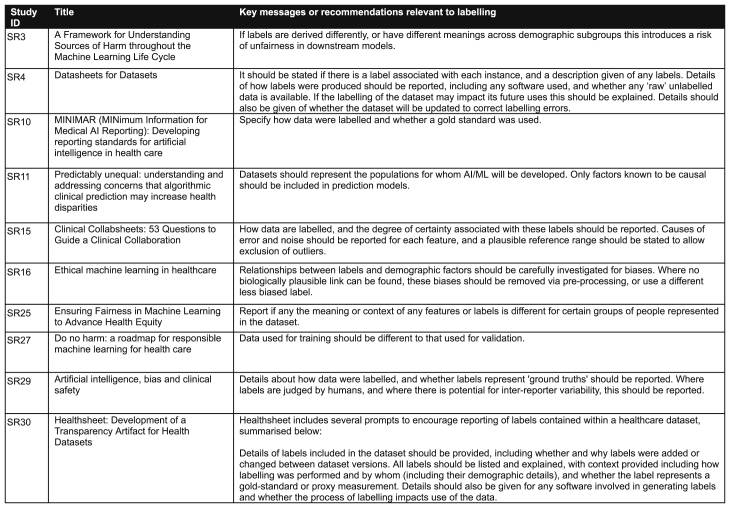
Key messages or recommendations relevant to labeling

Key messages or recommendations extracted from each source relating to data labeling and how this may be a cause of bias.

**Extended Data Table 4:**
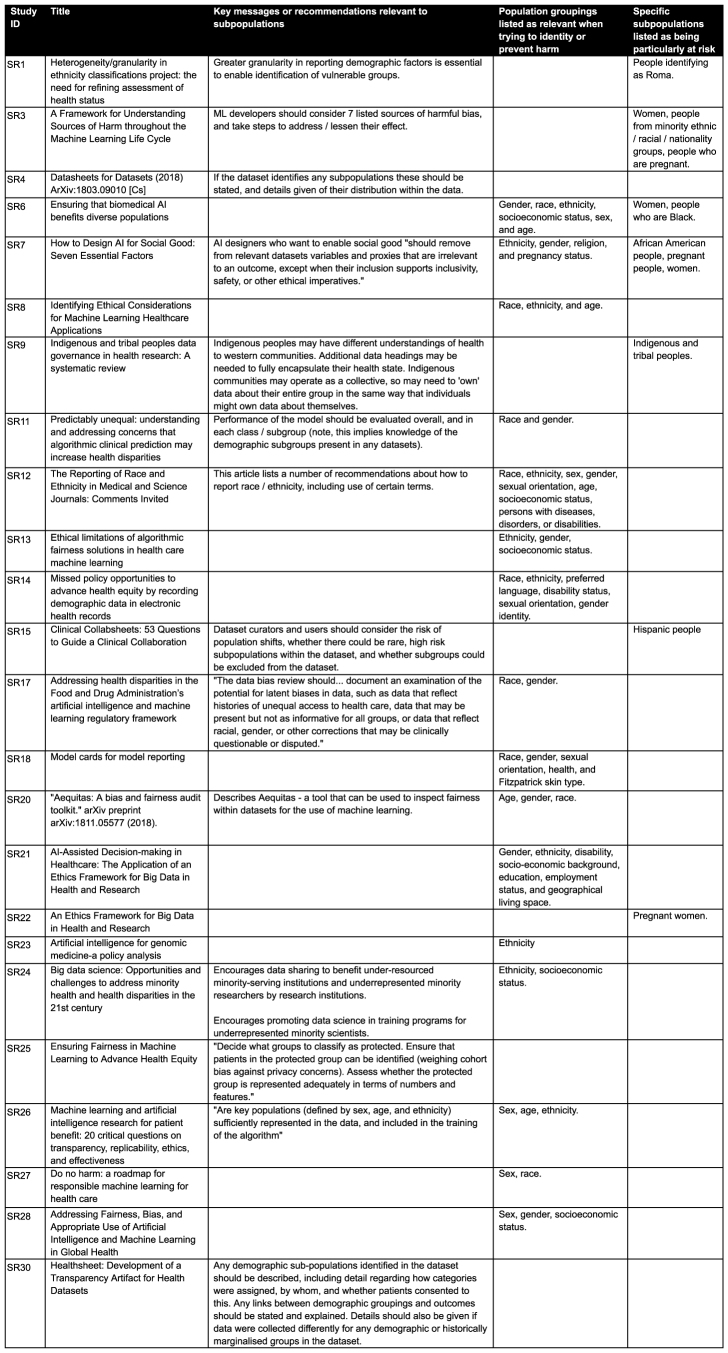
Key messages or recommendations relevant to subpopulations

Key messages or recommendations extracted from each source relating to subpopulations contained in datasets, including any steps that should be taken to reduce the risk of algorithmic biases for these groups. Also summarized are any subpopulations listed in each source as being particularly at risk of harm caused by algorithmic biases.

## Data Availability

All relevant data are included in the manuscript and supplementary files. Reproducible searches for Web of Science (https://webofscience.com/), Ovid MEDLINE (through ovid.com) and Embase (through ovid.com) are also included in the Methods, with relevant direct links.

## Code availability

No computer code was used. Qualtrics XM (https://www.qualtrics.com/uk/) was used to support the survey. NVivo was used to help refine the initial list of codes from the survey to create a codebook (https://www.qsrinternational.com/nvivo-qualitative-data-analysis-software/home).

## References

[1] Sidey-Gibbons JAM, Sidey-Gibbons CJ (2019). Machine learning in medicine: a practical introduction. BMC Med. Res. Methodol..

[2] Ibrahim H, Liu X, Zariffa N, Morris AD, Denniston AK (2021). Health data poverty: an assailable barrier to equitable digital health care. Lancet Digit. Health.

[3] Kuhlman, C., Jackson, L. & Chunara, R. No computation without representation: avoiding data and algorithm biases through diversity. In *Proc. 26th ACM SIGKDD International Conference on Knowledge Discovery & Data Mining (KDD '20)* 3593 (ACM, 2020); 10.1145/3394486.3411074

[4] Courbier S, Dimond R, Bros-Facer V (2019). Share and protect our health data: an evidence based approach to rare disease patients’ perspectives on data sharing and data protection - quantitative survey and recommendations. Orphanet J. Rare Dis..

[5] Chen IY (2021). Ethical machine learning in healthcare. Annu Rev. Biomed. Data Sci..

[6] Khan SM (2021). A global review of publicly available datasets for ophthalmological imaging: barriers to access, usability, and generalisability. Lancet Digit. Health.

[7] Wen D (2022). Characteristics of publicly available skin cancer image datasets: a systematic review. Lancet Digit. Health.

[8] Kaushal A, Altman R, Langlotz C (2020). Geographic distribution of US cohorts used to train deep learning algorithms. JAMA.

[9] Gichoya JW (2022). AI recognition of patient race in medical imaging: a modelling study. Lancet Digit. Health.

[10] Glocker, B., Jones, C., Bernhardt, M. & Winzeck, S. Risk of bias in chest radiography foundation models. *Radiol. Artif. Intell.*10.1148/ryai.230060 (2023). 10.1148/ryai.230060 PMC10698597 38074789

[11] Zou, J. & Schiebinger, L. Ensuring that biomedical AI benefits diverse populations. *eBioMedicine*10.1016/j.ebiom.2021.103358 (2021). 10.1016/j.ebiom.2021.103358 PMC8176083 33962897

[12] Jobin A, Ienca M, Vayena E (2019). The global landscape of AI ethics guidelines. Nat. Mach. Intell..

[13] *Ethics and Governance of Artificial Intelligence for Health* (WHO 2021); https://www.who.int/publications-detail-redirect/9789240029200

[14] Block RG (2020). Recommendations for improving national clinical datasets for health equity research. J. Am. Med. Inform. Assoc..

[15] DeVoe JE (2014). The ADVANCE network: accelerating data value across a national community health center network. J. Am. Med. Inform. Assoc..

[16] Hasnain-Wynia R, Baker DW (2006). Obtaining data on patient race, ethnicity, and primary language in health care organizations: current challenges and proposed solutions. Health Serv. Res..

[17] *Computer-Assisted Detection Devices Applied to Radiology Images and Radiology Device Data - Premarket Notification [510(k)] Submission*s. (FDA, 2022); https://www.fda.gov/regulatory-information/search-fda-guidance-documents/computer-assisted-detection-devices-applied-radiology-images-and-radiology-device-data-premarket

[18] Ganapathi S (2022). Tackling bias in AI health datasets through the STANDING Together initiative. Nat. Med..

[19] Vollmer S (2020). Machine learning and artificial intelligence research for patient benefit: 20 critical questions on transparency, replicability, ethics, and effectiveness. Br. Med. J..

[20] Challen R (2019). Artificial intelligence, bias and clinical safety. BMJ Qual. Saf..

[21] Wiens J (2019). Do no harm: a roadmap for responsible machine learning for health care. Nat. Med..

[22] Saleh, S., Boag, W., Erdman, L. & Naumann, T. Clinical collabsheets: 53 questions to guide a clinical collaboration. In *Proc.**5th Machine Learning for Healthcare Conference* (eds Doshi-Velez, F. et al.) 783–812 (PMLR, 2022); https://proceedings.mlr.press/v126/saleh20a.html

[23] Ferryman K (2020). Addressing health disparities in the Food and Drug Administration’s artificial intelligence and machine learning regulatory framework. J. Am. Med. Inform. Assoc..

[24] Suresh, H. & Guttag, J. A framework for understanding sources of harm throughout the machine learning life cycle. In *Proc. Equity and Access in Algorithms, Mechanisms, and Optimization* 1–9 (ACM, 2021); https://dl.acm.org/doi/10.1145/3465416.3483305

[25] Lysaght T, Lim HY, Xafis V, Ngiam KY (2019). AI-assisted decision-making in healthcare: the application of an ethics framework for big data in health and research. Asian Bioeth. Rev..

[26] Flanagin A, Frey T, Christiansen SL, Bauchner H (2021). The reporting of race and ethnicity in medical and science journals: comments invited. JAMA.

[27] Cerdeña JP, Grubbs V, Non AL (2022). Racialising genetic risk: assumptions, realities, and recommendations. Lancet.

[28] Elias, J. Google contractor reportedly tricked homeless people into face scans. *CNBC* https://www.cnbc.com/2019/10/03/google-contractor-reportedly-tricked-homeless-people-into-face-scans.html (2019).

[29] *Equality Act 2010. Statute Law Database* (UK Government, 2010); https://www.legislation.gov.uk/ukpga/2010/15/section/4

[30] *Declaration of the High-Level Meeting of the General Assembly on the Rule of Law at the National and International Levels* (UN General Assembly, 2012); https://digitallibrary.un.org/record/734369

[31] *Article 21 - Non-Discrimination* (European Union Agency for Fundamental Rights, 2007); https://fra.europa.eu/en/eu-charter/article/21-non-discrimination

[32] Gebru, T. et al. Datasheets for datasets. Preprint at http://arxiv.org/abs/1803.09010 (2021).

[33] Rostamzadeh, N. et al. Healthsheet: development of a transparency artifact for health datasets. In *Proc. 2022 ACM Conference on Fairness, Accountability, and Transparency* 1943–1961 (ACM, 2022); 10.1145/3531146.3533239

[34] Smeaton, J. & Christie, L. AI and healthcare. *UK Parliament POSTnote* https://post.parliament.uk/research-briefings/post-pn-0637/ (2021).

[35] Human bias and discrimination in AI systems. *ICO* https://webarchive.nationalarchives.gov.uk/ukgwa/20211004162239/https://ico.org.uk/about-the-ico/news-and-events/ai-blog-human-bias-and-discrimination-in-ai-systems/ (2019).

[36] *Artificial Intelligence and Machine Learning in Software as a Medical Device* (FDA, 2021); https://www.fda.gov/medical-devices/software-medical-device-samd/artificial-intelligence-and-machine-learning-software-medical-device

[37] *A Governance Framework for Algorithmic Accountability and Transparency* (European Parliament, Directorate-General for Parliamentary Research Services, 2019); https://data.europa.eu/doi/10.2861/59990

[38] *WHO Issues First Global Report on Artificial Intelligence (AI) in Health and Six Guiding Principles for Its Design and Use* (WHO, 2021); https://www.who.int/news/item/28-06-2021-who-issues-first-global-report-on-ai-in-health-and-six-guiding-principles-for-its-design-and-use

[39] *Regulatory Horizons Council: The Regulation of Artificial Intelligence as a Medical Device*. (UK Government, 2022); https://www.gov.uk/government/publications/regulatory-horizons-council-the-regulation-of-artificial-intelligence-as-a-medical-device

[40] Arora A, Arora A (2022). Generative adversarial networks and synthetic patient data: current challenges and future perspectives. Future Healthc. J..

[41] Burlina P, Joshi N, Paul W, Pacheco KD, Bressler NM (2021). Addressing artificial intelligence bias in retinal diagnostics. Transl. Vis. Sci. Technol..

[42] Koivu A, Sairanen M, Airola A, Pahikkala T (2020). Synthetic minority oversampling of vital statistics data with generative adversarial networks. J. Am. Med. Inform. Assoc..

[43] Murphy K (2021). Artificial intelligence for good health: a scoping review of the ethics literature. BMC Med. Ethics.

[44] Wilkinson MD (2016). The FAIR guiding principles for scientific data management and stewardship. Sci. Data.

[45] Liu X, Cruz Rivera S, Moher D, Calvert MJ, Denniston AK (2020). Reporting guidelines for clinical trial reports for interventions involving artificial intelligence: the CONSORT-AI extension. Nat. Med..

[46] Villarroel N, Davidson E, Pereyra-Zamora P, Krasnik A, Bhopal RS (2019). Heterogeneity/granularity in ethnicity classifications project: the need for refining assessment of health status. Eur. J. Public Health.

[47] Denton, E. et al. Bringing the people back in: contesting benchmark machine learning datasets. Preprint at http://arxiv.org/abs/2007.07399 (2020).

[48] Holland, S., Hosny, A., Newman, S., Joseph, J. & Chmielinski, K. The dataset nutrition label: a framework to drive higher data quality standards. Preprint at http://arxiv.org/abs/1805.03677 (2018).

[49] Floridi L, Cowls J, King TC, Taddeo M (2020). How to design AI for social good: seven essential factors. Sci. Eng. Ethics.

[50] Char DS, Abràmoff MD, Feudtner C (2020). Identifying ethical considerations for machine learning healthcare applications. Am. J. Bioeth..

[51] Griffiths KE, Blain J, Vajdic CM, Jorm L (2021). Indigenous and tribal peoples data governance in health research: a systematic review. Int. J. Environ. Res. Public Health.

[52] Hernandez-Boussard T, Bozkurt S, Ioannidis JPA, Shah NH (2020). MINIMAR (MINimum Information for Medical AI Reporting): developing reporting standards for artificial intelligence in health care. J. Am. Med. Inform. Assoc..

[53] Paulus JK, Kent DM (2020). Predictably unequal: understanding and addressing concerns that algorithmic clinical prediction may increase health disparities. NPJ Digit. Med..

[54] McCradden MD, Joshi S, Mazwi M, Anderson JA (2020). Ethical limitations of algorithmic fairness solutions in health care machine learning. Lancet Digit. Health.

[55] Douglas MD, Dawes DE, Holden KB, Mack D (2015). Missed policy opportunities to advance health equity by recording demographic data in electronic health records. Am. J. Public Health.

[56] Mitchell, M. et al. Model cards for model reporting. In *Proc. Conference on Fairness, Accountability, and Transparency (FAT* '19*) 220–229 (ACM, 2019); 10.1145/3287560.3287596

[57] Mörch CM, Gupta A, Mishara BL (2020). Canada protocol: an ethical checklist for the use of artificial Intelligence in suicide prevention and mental health. Artif. Intell. Med..

[58] Saleiro, P. et al. Aequitas: a bias and fairness audit toolkit. Preprint at http://arxiv.org/abs/1811.05577 (2019).

[59] Xafis V (2019). An ethics framework for big data in health and research. Asian Bioeth. Rev..

[60] Abstracts from the 53rd European Society of Human Genetics (ESHG) conference: e-posters. *Eur. J. Hum. Genet.* **28**, 798–1016 (2020). 10.1038/s41431-020-00741-5 PMC7705408 33262486

[61] Zhang X (2017). Big data science: opportunities and challenges to address minority health and health disparities in the 21st century. Ethn. Dis..

[62] Rajkomar A, Hardt M, Howell MD, Corrado G, Chin MH (2018). Ensuring fairness in machine learning to advance health equity. Ann. Intern. Med..

[63] Fletcher, R. R., Nakeshimana, A. & Olubeko, O. Addressing fairness, bias, and appropriate use of artificial intelligence and machine learning in global health. *Front. Artif. Intell.*10.3389/frai.2020.561802 (2021). 10.3389/frai.2020.561802 PMC8107824 33981989

[64] The PRISMA 2020 statement: an updated guideline for reporting systematic reviews. *BMJ* **372**, n71 (2021). 10.1136/bmj.n71 PMC8005924 33782057

[65] Braun V, Clarke V (2006). Using thematic analysis in psychology. Qual. Res. Psychol..

