# Supplementary material for: The value of standards for health datasets in artificial intelligence-based applications
Source: Nat Med. 2023 Oct 26;29(11):2929–38. doi: 10.1038/s41591-023-02608-w (PMC10667100; doi:10.1038/s41591-023-02608-w)
Supplement: Supplementary file 1 — Scoping survey questions. [file 41591_2023_2608_MOESM1_ESM.pdf]

nature medicine

Analysis

https://doi.org/10.1038/s41591-023-02608-w

The value of standards for health datasets in artificial intelligence-based applications

In the format provided by the authors and unedited

STANDING Together

STANDING Together - stakeholder survey

STANDING Together - a project to develop STANdards for data Diversity, INclusivity and Generalisability.

We would like to invite you to participate in a survey on recommendations around diversity of health data for medical AI algorithms. The aim of this study is to develop standards for health data, used to support development of AI algorithms to ensure that these algorithms do not disadvantage minority population groups.

If you have relevant experience in health data science, machine learning, health inequalities research, we would like to hear from you. The online survey will take approximately 20 minutes to complete. Further details are provided on the next page.

Participant Information Sheet (Online survey)

Study Title: Developing STANdards for data Diversity, INclusivity and Generalisability (STANDING together)

WHAT IS THE PURPOSE OF THIS STUDY?
Artificial Intelligence provides a platform that can make accessible and personalised medicine a reality. Whilst AI has the potential to improve healthcare, it can also perpetuate and even exacerbate existing societal biases leading to further health inequalities. A key consideration is representativeness of the data underpinning AI: if the data is not representative of specific populations, there is a risk that the AI algorithm will underperform in those individuals. Poor data composition and quality in underrepresented groups risks poorer algorithmic performance and perpetuation of societal biases.

To address this, gathering of health data needs to be designed with inclusivity and diversity in mind. We need standards to guide how AI datasets should be composed (‘who’ is represented in the data) and transparency around the data composition (‘how’ they are represented).

This project is about developing standards for health data, to support development of AI algorithms which do not disadvantage minoritised population groups. The standards arising from this project will be of importance to policy makers, regulators, developers of AI systems, patients and healthcare professionals.

WHO IS ORGANISING AND FUNDING THE RESEARCH?
This project is being conducted by an international team of researchers from multiple research institutes – the full list of co-investigators can be found at www.datadiversity.org . The study is funded by the National Institute of Health Research, NHSx and The Health Foundation as part of an AI and Racial and Ethnic Inequalities in Health and Care award (AI_HI200014).

WHY ARE WE APPROACHING YOU?
We are inviting individuals who have expertise in machine learning, health data science, digital health technologies and health inequalities. Your participation will help us to identify the items that should be incorporated in the standards we are seeking to produce.

WHAT WILL HAPPEN TO ME IF I TAKE PART?
You can access the online survey directly via the link provided in the invitation email. The survey will take approximately 15-30 minutes to complete.

DO I HAVE TO TAKE PART?
No, your participation is entirely voluntary. If you do decide to take part you will be given a copy of this information sheet and be asked to acknowledge consent via the online survey.

WHAT WILL HAPPEN IF I NO LONGER WANT TO TAKE PART IN THE STUDY?
You may change your mind at any time (before the start of the study or even after you have commenced the study) for whatever reason without having to justify your decision. However, as responses to this survey are being conducted anonymously we will not be able to remove your responses once they are submitted – it is therefore important that you do not press the 'submit' button unless you are content for your responses to be stored. If you choose to provide an email address or any other personal information at the end of the survey we will store this separately from your responses after submission. You may ask for the removal of the email address or any other personal information you have provided at any time. Please contact the research team (Dr Xiaoxuan Liu, x.liu.8@bham.ac.uk) and they will remove this information from the study database.

WHAT ARE THE POSSIBLE BENEFITS OF TAKING PART?
There will be no direct benefits for you if you decide to take part. However, there will be wider benefits anticipated for marginalised or disadvantaged groups, who may not have been previously represented in the development of AI in healthcare.

WILL MY TAKING PART IN THIS STUDY BE KEPT CONFIDENTIAL?
All information about you which is connected with the research study will be kept strictly confidential. Only the research team will have access to any data generated from this study. The data that we will collect from you include the following: name, contact details (email), age, gender, ethnicity, professional role, organisation you work for and geographical location. When we use your information for research, we rely on Article 6(1)e ("processing is necessary for the performance of a task carried out in the public interest") and Article 9(2)j ("processing is necessary for archiving purposes in the public interest, scientific or historical research purposes") of the General Data Protection Regulation (GDPR) in combination with Schedule 1, Part 1, Art 4 Data Protection Act (DPA) 2018. For more information on how we manage your data and what are your rights, our privacy notice can be accessed at
https://www.birmingham.ac.uk/privacy/index.aspx

Electronic data will be stored on either the University Hospitals Birmingham NHS

Foundation Trusts or University of Birmingham encrypted computers that are password-protected, whilst any data collected on paper, such as paper consent forms and any paper correspondence will be stored in locked filing cabinets in the researcher's office. Data will be kept for 10 years and after this retention period has finished, the study data will be destroyed in line with University of Birmingham's standard operating procedures. Any information used in publications or reports will be anonymised so that your identity cannot be known.

WHO HAS REVIEWED THIS STUDY?
This research study has been reviewed and approved by the University of Birmingham ethics committee (ERN_21-1831).

If you have concerns about any aspect of this study, or if you wish to withdraw, please contact Dr Xiaoxuan Liu (contact@datadiversity.org)

Phone: (+44) 0121 371 8132
Postal mail: STANDING Together, 2nd floor Office 10, Institute for Translational Medicine, Heritage Building, Mindelsohn Way, Birmingham, B15 2TH.

I confirm that I have read the participant information, and I agree to my responses being recorded for this study

- ○ Yes
- ○ No

I understand that my responses will be stored anonymously, so it will not be possible to remove them once I press the submit button

- ○ Yes
- ○ No

I understand that if I choose to provide an email address or contact information this will be stored separately to my responses. I can ask for my contact information to be removed at any time by contacting the research

team using the contact email addresses

- Yes
- No

Section 1: tell us about yourself

All questions are completely voluntary, and information you provide will be stored anonymously.

What is your ethnic group?

White

- English / Welsh / Scottish / Northern Irish / British
- Irish
- Gypsy or Irish Traveller
- Any other White background (please describe below)

Mixed / multiple ethnic groups

- White and Black Caribbean
- White and Black African
- White and Asian
- Any other Mixed / Multiple ethnic background (please describe below)

Asian / Asian British

- Indian
- Pakistani
- Bangladeshi
- Chinese
- Any other Asian background (please describe below)

Black / African / Caribbean / Black British

- African
- Caribbean
- Any other Black / African / Caribbean background (please describe below)

Other ethnic group

- Arab
- Any other ethnic group (please describe below)

Please describe your ethnic group

What is your sex?

- Male
- Female
- Prefer not to say

Is the gender you identify with the same as your sex registered at birth?

This question is voluntary

- Yes
- No
- Prefer not to say

What is your profession and expertise?

In which country are you based?

Section 2: how big is the issue?

Don't worry if you don't feel able to write a response to each question - we suggest you focus your attention on topics that resonate with you the strongest.

Do you think demographic standards (in particular, ethnic / racial) for AI datasets are needed, and why?

Do you know of existing standards for healthcare datasets?

Section 3: what is your experience?

Don't worry if you don't feel able to write a response to each question - we suggest you focus your attention on topics that resonate with you the strongest.

In your own work with medical AI algorithms, how do you use demographic information? Were there any specific challenges?

How did you address any challenges you encountered?

Section 4: what would a diverse dataset look like?

Don't worry if you don't feel able to write a response to each question - we suggest you focus your attention on topics that resonate with you the strongest.

What makes a healthcare dataset diverse?

Is 'diversity' a universal concept, or should it be different for different contexts? How do we define what diversity should look like in each context?

How do we decide whether demographics such as race and ethnicity matters to medical AI models?

Is it possible to develop a set of metrics to rate datasets on demographic diversity? What could these metrics measure?

Can dataset curators self-report these metrics, or does it need to be externally validated?

Are there unintended consequences of mandating standards?

Section 5: the practicality of operationalising standards.

Don't worry if you don't feel able to write a response to each question - we suggest you focus your attention on topics that resonate with you the strongest.

Regulators such as the FDA already recommend AI developers demonstrate performance across demographic subgroups, but it's not clear whether this is being done. What further steps can we take to ensure standards are followed?

How can we incentivise adoption of such standards in development and validation of medical AI algorithms?

Who should be responsible for ensuring / checking such standards are followed?

Section 6: closing questions

Is there anything else we should consider regarding this topic - and should pursue in our next round of interviews?

Powered by Qualtrics
