# Supplementary material for: The value of standards for health datasets in artificial intelligence-based applications
Source: Nat Med. 2023 Oct 26;29(11):2929–38. doi: 10.1038/s41591-023-02608-w (PMC10667100; doi:10.1038/s41591-023-02608-w)
Supplement: Supplementary file 2 — Reporting Summary [file 41591_2023_2608_MOESM2_ESM.pdf]

nature portfolio

Corresponding author(s): Dr Anmol Arora, Dr Xiaoxuan Liu

Last updated by author(s): Aug 25, 2023

Reporting Summary

Nature Portfolio wishes to improve the reproducibility of the work that we publish. This form provides structure for consistency and transparency in reporting. For further information on Nature Portfolio policies, see our Editorial Policies and the Editorial Policy Checklist.

Statistics

For all statistical analyses, confirm that the following items are present in the figure legend, table legend, main text, or Methods section.

n/a	Confirmed	
☐	☒	The exact sample size (*n*) for each experimental group/condition, given as a discrete number and unit of measurement
☒	☐	A statement on whether measurements were taken from distinct samples or whether the same sample was measured repeatedly
☒	☐	The statistical test(s) used AND whether they are one- or two-sided<br>*Only common tests should be described solely by name; describe more complex techniques in the Methods section.*
☒	☐	A description of all covariates tested
☒	☐	A description of any assumptions or corrections, such as tests of normality and adjustment for multiple comparisons
☒	☐	A full description of the statistical parameters including central tendency (e.g. means) or other basic estimates (e.g. regression coefficient) AND variation (e.g. standard deviation) or associated estimates of uncertainty (e.g. confidence intervals)
☒	☐	For null hypothesis testing, the test statistic (e.g. *F*, *t*, *r*) with confidence intervals, effect sizes, degrees of freedom and *P* value noted<br>*Give P values as exact values whenever suitable.*
☒	☐	For Bayesian analysis, information on the choice of priors and Markov chain Monte Carlo settings
☒	☐	For hierarchical and complex designs, identification of the appropriate level for tests and full reporting of outcomes
☒	☐	Estimates of effect sizes (e.g. Cohen's *d*, Pearson's *r*), indicating how they were calculated

Our web collection on statistics for biologists contains articles on many of the points above.

Software and code

Policy information about availability of computer code

Data collection	No computer code was used. Qualtrics XM (https://www.qualtrics.com/uk/) was used to support the survey.
Data analysis	No computer code was used. NVivo was used to help refine the initial list of codes from the survey to create a codebook (https://www.qsrinternational.com/nvivo-qualitative-data-analysis-software/home)

For manuscripts utilizing custom algorithms or software that are central to the research but not yet described in published literature, software must be made available to editors and reviewers. We strongly encourage code deposition in a community repository (e.g. GitHub). See the Nature Portfolio guidelines for submitting code & software for further information.

Data

Policy information about availability of data

All manuscripts must include a data availability statement. This statement should provide the following information, where applicable:

- Accession codes, unique identifiers, or web links for publicly available datasets
- A description of any restrictions on data availability
- For clinical datasets or third party data, please ensure that the statement adheres to our policy

All relevant data is included within the manuscript and supplementary files. Reproducible searches for Web of Science (https://webofscience.com/), OVID MEDLINE (via ovid.com) and EMBASE (via ovid.com) are also included in the Methods, with relevant direct links.

Human research participants

Policy information about studies involving human research participants and Sex and Gender in Research.

Reporting on sex and gender	Data analysis of survey responses was not disaggregated by sex or gender identity because the qualitative analysis approach (inductive, exploratory) was not intended to identify differences between sex / gender groups.
Population characteristics	Twenty participants completed the scoping survey. Of these participants, 10 (50%) reported their sex as Female, 9 (45%) reported their sex as Male, and 1 (5%) did not provide this information. 18 participants (90%) reported their gender identity was the same as the sex registered at birth, one participant (5%) reported that their gender identity was different to their sex registered at birth, and one participant (5%) did not provide this information.
Recruitment	We took a broad approach to the recruitment of different types of stakeholders in the scoping survey and therefore did not apply a formal sampling framework. As a result, we cannot be sure that the voices represented are consistent across all stakeholders.
Ethics oversight	This research was conducted in compliance with all relevant ethical regulations, including informed consent from all participants. Ethical approval was granted by the University of Birmingham's Science, Technology, Engineering and Mathematics Ethical Review Committee (ERN_21-1831).

Note that full information on the approval of the study protocol must also be provided in the manuscript.

Field-specific reporting

Please select the one below that is the best fit for your research. If you are not sure, read the appropriate sections before making your selection.

☒ Life sciences ☐ Behavioural & social sciences ☐ Ecological, evolutionary & environmental sciences

For a reference copy of the document with all sections, see nature.com/documents/nr-reporting-summary-flat.pdf

Life sciences study design

All studies must disclose on these points even when the disclosure is negative.

Sample size	No statistical method was used to predetermine the sample size for the stakeholder survey. Twenty participants completed the scoping survey, covering a range of expertise. The sample size was determined pragmatically based on the availability of participants and investigators.
Data exclusions	Data relating to the impact of articles included in our systematic review (including journal impact factor, citation count and altmetric data) were obtained but not included in the analysis, because these data were not necessary to extract themes from the included articles.
Replication	Reproducible search strategies are included within the manuscript, with direct url links to relevant databases. A full reproducible methodology is provided for both the literature review and scoping survey.
Randomization	Randomization is not applicable to non-interventional studies.
Blinding	Blinding is not applicable to non-interventional studies.

Reporting for specific materials, systems and methods

We require information from authors about some types of materials, experimental systems and methods used in many studies. Here, indicate whether each material, system or method listed is relevant to your study. If you are not sure if a list item applies to your research, read the appropriate section before selecting a response.

Materials & experimental systems

n/a	Involved in the study	
☒	☐	Antibodies
☒	☐	Eukaryotic cell lines
☒	☐	Palaeontology and archaeology
☒	☐	Animals and other organisms
☒	☐	Clinical data
☒	☐	Dual use research of concern

Methods

n/a	Involved in the study	
☒	☐	ChIP-seq
☒	☐	Flow cytometry
☒	☐	MRI-based neuroimaging
